# Protocol for randomized controlled trial to evaluate the safety and feasibility of a novel helmet to deliver transcranial light emitting diodes photobiomodulation therapy to patients with Parkinson’s disease

**DOI:** 10.3389/fnins.2022.945796

**Published:** 2022-08-17

**Authors:** Claire McGee, Ann Liebert, Geoffrey Herkes, Brian Bicknell, Vincent Pang, Craig S. McLachlan, Hosen Kiat

**Affiliations:** ^1^Faculty of Health Sciences, Torrens University, Sydney, NSW, Australia; ^2^School of Medical Sciences, University of Sydney, Camperdown, NSW, Australia; ^3^Department of Research and Governance, San Hospital, Wahroonga, NSW, Australia; ^4^NICM Health Research Institute, University of Western Sydney, Westmead, NSW, Australia; ^5^Department of Neurology, San Hospital, Wahroonga, NSW, Australia; ^6^Australian National University, Canberra, ACT, Australia; ^7^Centre for Healthy Futures, Torrens University, Sydney, NSW, Australia; ^8^Faculty of Medicine, Human and Health Sciences, Macquarie University, Sydney, NSW, Australia; ^9^College of Health and Medicine, Australian National University, Canberra, ACT, Australia; ^10^Cardiac Health Institute, Sydney, NSW, Australia

**Keywords:** photobiomodulation, transcranial, Parkinson’s disease, cognitive dysfunction, mobility

## Abstract

**Introduction:**

Parkinson’s disease (PD) is the second most common, progressive, and debilitating neurodegenerative disease associated with aging and the most common movement disorder. Photobiomodulation (PBM), the use of non-thermal light for therapeutic purposes using laser or light emitting diodes (LED) is an emerging non-invasive treatment for a diverse range of neurological conditions. The main objectives of this clinical trial are to investigate the feasibility, safety, tolerability, and efficacy of a novel transcranial LED helmet device (the “PDNeuro”) in the alleviation of symptoms of PD.

**Methods and analysis:**

This is a 24-week, two-arm, triple-blinded randomized placebo-controlled clinical trial of a novel transcranial “PDNeuro” LED Helmet, comparing an active helmet to a sham helmet device. In a survey, 40 PD participants with Hoehn and Yahr Stage I–III during ON periods will be enrolled and randomly assigned into two groups. Both groups will be monitored weekly for the safety and tolerability of the “PDNeuro” LED Helmet. Clinical signs and symptoms assessed will include mobility, fine motor skills and cognition, with data collected at baseline, 12 weeks, and 24 weeks. Assessment tools include the TUG, UPDRS, and MoCA all validated for use in PD patients. Patient’s adherence to the device usage and participant drop out will be monitored weekly. At 12 weeks both placebo and treatment groups will crossover and placebo participants offered the treatment. The main indicator for clinical efficacy of the “PDneuro” Helmet is evidence of sustained improvements in motor and non-motor symptoms obtained from participant self-reported changes, carer reporting of changes and objective reassessment by the investigators. The outcomes will assist in a future larger randomized trial design.

**Clinical Trial Registration:**

[https://www.anzctr.org.au], identifier [12621001722886].

## Background

Parkinson’s disease (PD) is a complex, heterogenous neurodegenerative disorder that presents with both motor and non-motor symptoms (DeMaagd and Philip, 2015). Cognitive decline can be a late manifestation of the disease and as these symptoms usually progress slowly, patients can be living for many years with impaired cognition (Mack and Marsh, 2017). Treatment for PD has often involved multi-modalities including pharmacotherapy, surgical intervention, and physiotherapy (Iarkov et al., 2020). Although significant inroads in PD treatment have been made, to date, optimal treatment for a range of motor, non-motor, and non-dopaminergic symptoms remains a major therapeutic challenge (Hayes et al., 2019).

Consequently, identifying alternative non-invasive, therapeutic methods is needed. Photobiomodulation (PBM) is a non-invasive treatment modality that allows wavelengths of red or near-infrared light to reach tissues beyond the surface of the skin. This therapy has been demonstrated to produce a range of beneficial physiological changes (Heiskanen and Hamblin, 2018). Various light sources, including lasers and light emitting diodes (LEDs) can be applied. PBM therapy (PBMt) was traditionally delivered with hand-held lasers and more recently *via* LED devices, for a wide variety of conditions (Heiskanen and Hamblin, 2018).

To affect the brain using an LED transcranial device, the light must penetrate the skin and reach the tissues of the brain with a sufficient dose to interact with neurons in the brain. The effectiveness of PBMt is therefore dependent on the amount and rate of light energy that can penetrate the human scalp and skull. PBM penetration to the brain has been investigated with results suggesting that near infrared (NIR) light can penetrate to varying depths (Haeussinger et al., 2011; Tedford et al., 2015; Henderson and Morries, 2019; Salehpour et al., 2019). Salehpour et al. (2019), performed a review of the literature and found that on average, penetration of transcranial red/NIR (630–810 nm) is between 0.2 and 10% in humans. In addition, in a cadaver model, transcranial light at 808 nm wavelength has been demonstrated to penetrate the human skull to a depth of 40 mm (Tedford et al., 2015). Haeussinger et al. (2011) described a mean penetration depth of approximately 23 mm. As well, using human cadaver heads (formalin fixed), light has been shown to reach brain parenchyma with the percent penetrance ranging from 12% in the occipital region and 1% in the temporal region (Jagdeo et al., 2012). Used as a proxy for human head, studies on sheep skull showed that at 10–15 W power range, 810 and 980 nm NIR can provide biologically meaningful depth penetration of 30 mm (Henderson and Morries, 2015).

A sleuth of literature exists on the direct effects of PBMt on neuronal cellular metabolism (Hamblin, 2018; Dompe et al., 2020; Hamblin and Liebert, 2022; Wu et al., 2022) with light absorption within the mitochondrial electron transport chain increasing ATP production, leading to a reduction in neuronal death, reduced neuroinflammation, increased cell survival and down regulation of proinflammatory markers.

Specifically, the effect of transcranial LED’s on brain function has been well-studied with changes to various parameters noted in animal models and humans (Shaw et al., 2010; Naeser and Hamblin, 2011; Jahan et al., 2019; Zomorrodi et al., 2019; Longo et al., 2020; El Khoury et al., 2021; Yao et al., 2021). The effects reported in human studies include increased activity in areas associated with attention and novelty (El Khoury et al., 2021), beneficial effects in attentional performance (Jahan et al., 2019), positive effects on modulating brain activity (Yao et al., 2021), increased organization of neural function (Zomorrodi et al., 2019) and a beneficial effect on myelin repair pathways (Longo et al., 2020).

There are also studies that demonstrate improvement in neurological symptoms in patients with PD and other conditions when treated with transcranial PBM. Light therapy in PD has been found to be of clinical benefit in a PD case series (Hamilton et al., 2019). This and other case studies using investigational PBMt devices on neural and cognitive function have presented promising results such as increased energy (Hamblin and Huang, 2019), improved gait and cognition (Saltmarche et al., 2017; Vargas et al., 2017), improved speech, and reduction in freezing episodes (Maloney et al., 2010). A small pilot double blind, placebo-controlled trial (*n* = 11) that examined the effect of transcranial PBMt on mild cognitive dysfunction also reported favorable clinical outcomes (Berman and Nichols, 2019). Recently, Nizamutdinov et al. (2021) demonstrated improvement across several brain functions in dementia patients treated with transcranial LED when compared with a sham treated group. A recent small study on PBMt delivered *via* a transcranial device on PD patients documented its safety and tolerability and demonstrated measurable improvements in several PD related signs and symptoms (Liebert et al., 2021). Transcranial helmet devices appear an ideal solution and an important mode of delivery for PBMt due to ease of self-application and ability to irradiate large area of tissue (Heiskanen and Hamblin, 2018).

**FIGURE 1:**
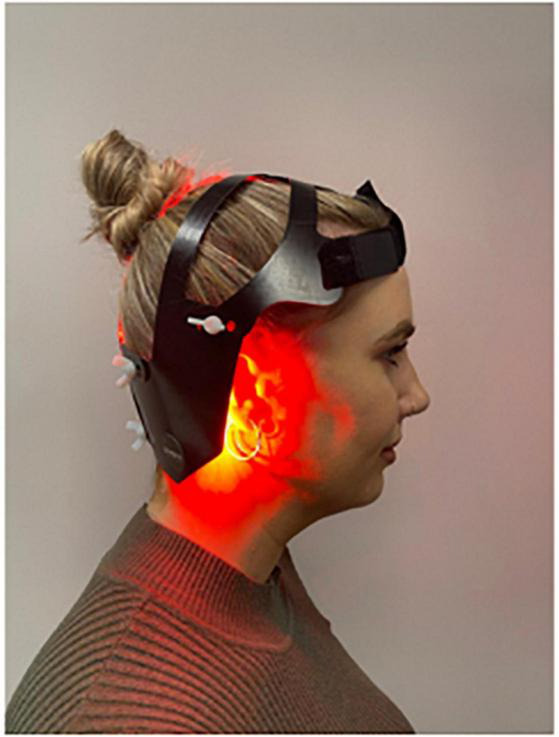
PDNeuro posterior view.

**FIGURE 2:**
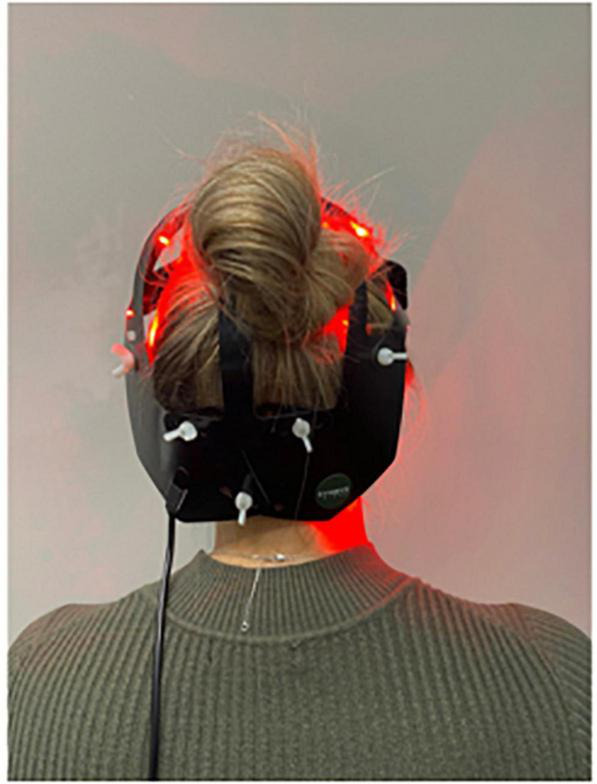
PDNeuro lateral view.

The “PDNeuro” Helmet (see Figures 1, 2) allows transcranial application of light at 20 locations, with each location including one infra-red (IR) 810 nm LED and one red 630–670 nm LED. The locations of the “PDNeuro” Helmet LEDs were selected based on anatomic points that have been used clinically and in published studies on neurodegenerative disease (Saltmarche et al., 2017; Hamilton et al., 2019; Liebert et al., 2021). The points include the mastoid process and the second cervical vertebrae points of the sub-occipital region. They are intended to target the corresponding dermatome to permit sensory input to converge onto the trigeminocervical nuclei and the neural connections of the brain stem and cerebellum pathways, including the putative endorestiform nucleus (Bradman and Barry, 2013; Paxinos et al., 2020).

This triple blinded, randomized controlled, at-home 24-week trial (RCT) with a crossover of the placebo group, has been designed to test the safety and effectiveness of the “PDNeuro” Helmet device in modifying clinical signs and symptoms experienced by PD patients. This compliments a previous experimental study that showed efficacy of a combined treatment protocol (transcranial with an abdominal, neck, and nasal application) in PD patients (Liebert et al., 2021). In previous published studies, the authors have demonstrated observable changes after 4-weeks of intervention, with statistical significance reported at 12-weeks utilizing both transcranial and remote PBMt, suggesting that 24-weeks will be sufficient to demonstrate significant effects with the present study being a randomized controlled trial with increased total number of participants (Liebert et al., 2021).

### Hypothesis and aims

The main objectives of the study described here are to; investigate the feasibility, safety, tolerability, and efficacy of a novel transcranial LED helmet device (the “PDNeuro” Helmet) and determine the therapeutic effects of PBMt when applied 6 days per week over 12 weeks.

### Hypotheses

1. Primary Hypothesis: Transcranial LED PBMt is a safe, non-invasive therapeutic intervention for patients with diagnosed PD.
2. Secondary Hypothesis: Treatment by Transcranial LED PBMt attenuates motor and non-motor symptoms of PD.

## Methods and analysis

### Trial design

**FIGURE 3:**
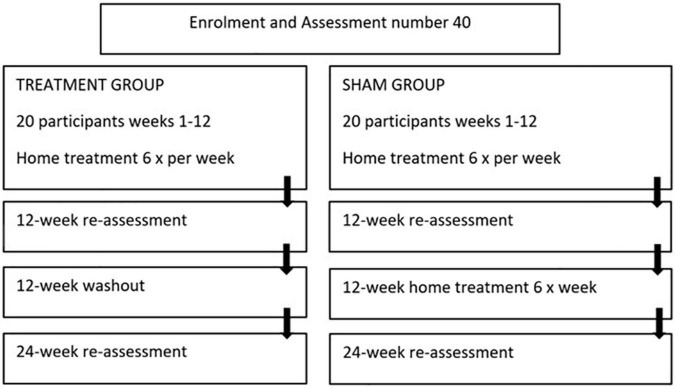
Protocol for recruitment selection and initial screening and baseline data collection.

The study is a 24-week, two-arm, triple blinded randomized placebo-controlled trial evaluating the safety and effectiveness of the “PDNeuro” Helmet (see Figures 1, 2) compared with a sham helmet device. Treatment will consist of PBMt to the head six times per week for 12 weeks (see Figure 3), with Group 1 (the active group) receiving 12 min of infra-red and 12 min red LED, and Group 2 (the sham group) receiving sham treatment for 24 min. After the initial 12 weeks, the sham patients will be given the active treatment for 12 weeks while the active treatment group will have a 12 weeks washout period and then be re-assessed in 12 weeks (see Figure 3). Outcome measures will be collected by the study investigators that have received specialized training in assessment and data collection.

The study received ethics approval from Sydney Adventist Health (SAH) human ethics research committee (HREC) (approval number 2019-032). All experiments are carried out in accordance with the approved ethics guidelines.

### Eligibility and recruitment

The study consists of 40 PD patients (20 male + 20 female) recruited *via* media advertisements on TV and local media.

**FIGURE 4:**
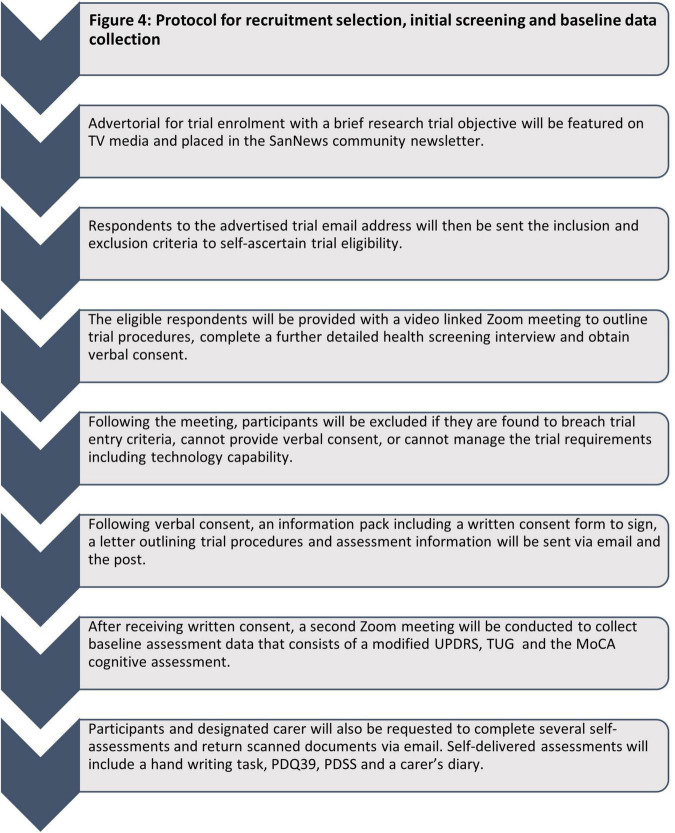
Protocol for treatment and assessments.

Due to the remote (virtual) nature of this trial, only PD participants categorized as Hoehn and Yahr Stage I-III during ON periods, and willing to have a carer present during all assessments and treatment sessions are included. Consent forms are signed by hand, and both emailed and posted back (see Figure 4 for overview of recruitment process).

### Inclusion criteria

• Females and males aged 59–85 years
• Diagnosed with Idiopathic PD (by United Kingdom Brain Bank Criteria) with Modified Hoehn and Yahr Stage I–III during ON periods
• ≥3 weeks of stable anti-PD medications
• Sufficient space (around 9 m^2^) to be able to perform motor assessments
• Suitable and sufficiently fast home-based internet connection for uninterrupted video calls and video conferencing
• Knowledge (self or carer) of using a phone and/or tablet applications on either IOS or Android platforms
• Attendance of a “carer” during each Zoom meeting and during all participant treatment sessions

Exclusion criteria – Participants will be excluded from the study if they:

• Are not capable of self-care
• History of significant psychotic episode(s) within the previous 12 months
• History of suicidal ideation or attempted suicide within previous 12 months
• Take potentially photosensitizing medications, in particular imipramine, hypericum, phenothiazine, lithium, chloroquine, hydrochlorothiazide, or tetracycline
• Have history of structural brain disease, active epilepsy, stroke, factors affecting gait performance and stance unrelated to PD, such as due to severe joint disease, orthopedic injuries, weakness, peripheral neuropathy with proprioceptive deficits, severe peripheral vascular occlusive disease, severe musculoskeletal disorders, uncorrected vision, vestibular problems or other acute illness or severe condition that would:
  - Preclude the use of PBM therapy
  - Place the patient at risk during evaluation of their PD, or
  - Interfere with the evaluation of their PD
• Are currently participating in other clinical trials, including treatment of PD
• Are currently using any form of self-administered light therapy
• Have evidence of severe and unstable dysautonomia
• Have significant cardiac disease
  ⚬ Cardiac interventions (in the past 3-months)
  ⚬ Unstable arrhythmias (in the past 3-months)
  ⚬ Diagnosis of cardiac dysautonomia

### Randomization

Following virtual screening and informed consent, 40 participants will be randomized into two groups and then allocated into a treatment or sham group *via* an independent research administrator and thereafter identified *via* an anonymous SN (serial number). To reduce the chance of participants inadvertently realizing that they are in the sham group, the HREC approved that the participants be informed there are four groups: active infrared, inactive infrared, active red + infrared, inactive red + infrared. Group 1 will receive active treatment and Group 2 will receive a sham helmet, which is identical in appearance. Participants will be instructed on helmet use by a trained technician *via* Zoom video communication.

By intention there will be equal numbers of males and females in each group. While it is well-known that gender differences effect human disease including PD, little research has been performed into the different gender responses to PBMt (Liebert et al., 2022). Therefore, the efficacy and tolerability outcomes of PBMt transcranial delivery will be subject to sub analysis based on gender.

### Intervention

The participants will be mailed their “PDNeuro” or sham helmet with all treatment to be self-administered at home in the presence of their carer. Study participants will be provided with detailed written and visual instructions on correct helmet fitment and treatment protocol. This will be supplemented with a Zoom video consultation with a trained technician and scientist to ensure correct device fitment and operation. During this Zoom consultation the helmet will be viewed from the frontal, sagittal, and transverse plane to ensure correct fitment. Participants will be subsequently monitored on a weekly basis with a compulsory consultation with the same trained research personnel to ensure continued correct fitment of the study device and adherence to the study protocol. In addition, all participants will be encouraged to reach out to the research team outside of these monitorisation periods for any trial related queries *via* either phone or email which are monitored 24-h per day, 7-days per week.

The PBMt intervention will consist of six 24 min-treatment sessions per week for 12 weeks (see Figure 3). Participants in Group 1, the active treatment group, will receive transcranial light treatment with a “PDNeuro” LED Helmet device with 20 LED stimulus sites, each having one IR (810 nm) LED and one Red (630–670 nm) LED. Average optical power for the IR LED (810 nm) is 52 mW and for the RED LED (630–670 nm) 27 mW. Treatment dose will consist of 0.052 W × 720 s to total 37.44 joules for IR and 0.027 W × 720 s to total 19.44 joules for red. Therefore, with 37.44 and 19.44 joules delivered over 20 diodes a total of 1,137 joules will be administered per session.

Participants in the placebo/sham group (Group 2) will receive the same apparent treatment as Group 1, except that they will be “treated” with sham transcranial LED devices that deliver no light. After the 12 weeks the placebo group will be offered a further 12 weeks of treatment with the active “PDNeuro” Helmet.

### Outcome measures

Due to the broad clinical signs and symptoms of PD, a range of outcome measures were selected to determine functional improvement (Table 1). Many PD clinical rating scales have been developed since the 1960s that assess both motor and non-motor functions (Ramaker et al., 2002) and all outcome measures to be used in this study have been validated for use in PD remotely (Abdolahi et al., 2013; Stillerova et al., 2016). The primary outcome measures will be conducted and recorded using a combination of self-reported assessment and visual assessment obtained *via* Zoom video link. Outcome measures will be supervised, and data will be collected *via* specialist examiners and physiotherapists all trained in conducting each assessment. Outcome measures will be obtained at baseline, 12-week and 24-week intervals (see Figure 3).

**TABLE 1:** Outcome measurements.

Primary outcome measurements	Data collection tools
1. Safety of PDNeuro helmet device	24-h access to health personnel Weekly [or as needed] technical meetings
2. Motor assessment	MDS-UPDRS [Modified Part 3 Motor component]
3. Social experiences and difficulties	PDQ39
**Secondary outcome measures**	
1. Mobility	TUG [non-cognitive]
2. Cognition	MoCA
3. Writing task	Repeated sentence writing to assess letter size
4. Sleep habits	Parkinson’s disease sleep scale (PDSS)
5. Carer’s diary	Qualitative assessment of care-givers thoughts

#### Primary outcome measurements

Safety of the “PDNeuro” Helmet device, MDS-Unified Parkinson’s Disease Rating Scale (MDS-UPDRS) modified Part 3 and social experiences and difficulties as measured with the Parkinson’s disease-specific quality of life questionnaire, the PDQ-39.

#### Secondary outcome measurements

1. Movement mobility (TUG [non-cognitive])
2. Cognition (MoCA)
3. Writing task (assess writing size)
4. Sleep habits self-assessment (PDSS sleep scale)
5. Carer’s diary of daily living

##### UPDRS

The primary motor measure chosen is the motor assessment (Part 3) of the UPDRS as it is one of the most common movement assessment outcomes used in PD (Ivey et al., 2012), and demonstrates high internal consistency and inter-rater reliability while showing moderate construct validity (Rodriguez-Blazquez et al., 2017).

Furthermore, the UPDRS assessment has been shown to be clinically valid when conducted remotely (Schneider et al., 2020). Indeed, the feasibility, reliability and value of implementing remote telemedicine/video-based assessments for Parkinson’s disease patients for both research (Tarolli et al., 2020) and ongoing healthcare (Cubo et al., 2020) have been previously evaluated and deemed valid.

Sixteen of the eighteen items from the UPDRS motor were included. Rigidity was excluded as this is unable to be determined without manual assessment. As well, postural stability was not performed due to the potential risk of falling if performed without adequate supervision. In the UPDRS all items are scored on a scale from 0 (normal) to 4 (severe), and total scores obtained from the sum of the corresponding item scores, stage the severity of PD. These scores are to be collected using video link with attention being paid to positioning the camera and participant to ensure clear vision. The carer will be required to manipulate the camera to ensure that the participant is clearly visible. All assessors are trained in the administration of the UPDRS assessment tool to maximize the outcome measure.

##### PDQ39

It is well-documented that quality of life (QOL) plays an important role in patient self-efficacy, is instrumental for independent living and, in chronic conditions such as PD is frequently greatly impaired (Megari, 2013). The questionnaire tool PDQ39 is a self-administered questionnaire that assesses PD specific quality of health during a specific time-period. It assesses across eight life dimensions: mobility, ADL’s, emotional well-being, stigma, social support, cognition, communication, and bodily discomfort (Hagell and Nygren, 2007). The PDQ39 will be administered by the patient during the same three time points: pre-treatment, following 12 weeks and 6 months post trial commencement.

##### Timed up and go test

To measure patients’ functional mobility, timed up and go (TUG) test will be used. The TUG test is a validated and effective tool to assess the participants functional mobility and the gait related motor symptoms common in PD (da Silva et al., 2017). The TUG involves sequential motor tasks that incorporate arising from a chair, walking, and turning around all of which are affected by PD. All assessors are trained in the administration of the TUG.

##### Montreal cognitive assessment

Cognitive impairment is a significant non-motor symptom of PD with as many as 80% of patients exhibiting decline in function (Flores-Torres et al., 2021). A frequently used assessment tool is the Montreal Cognitive Assessment (MoCA) a brief tool that assesses several cognitive domains. The MoCA is validated for use in 55–85-year-olds and recommended as a scale for cognitive screening in PD (Chou et al., 2010; Fang et al., 2020). The assessment consists of a 30-point test on a single side of A4 paper and can be administered in 10 min. All assessors are trained in the administration of the MoCA.

##### Writing task

Micrographia is a common clinical feature associated with PD and a measure of PD progression (Smits et al., 2014; Hamilton et al., 2019). Participants will be asked to write “the quick brown fox jumps over the lazy dog” and sign their name ten times and note of potential change in size will be recorded over the same three time-points.

##### Carer’s diary

A carer’s diary can provide qualitative data on patient experiences, as well as note changes in clinical manifestations that are not observed in the clinical environment. This information in the areas of physical, cognitive, and emotional domains can reveal both positive and negative changes and may provide a more detailed picture of the effect of treatment.

### Safety considerations

Photobiomodulation is considered a safe treatment. In over 50 years of research on the effects of PBMt, there have been limited published results of harm when used within the correct dose window. Cassano et al. (2019) noted transitory and benign side effects of transcranial PBMt and Maiello et al. (2019) reported one case of severe headache, which led to discontinuation of treatment.

Dosing protocols for the PD trial have been based on both clinical experience by members of the research team and others (Dompe et al., 2020). To provide for patient safety, a suspected adverse event committee was formed consisting of the coordinating chief investigator, a neurologist from the research team, and an independent neurologist. A member of this team will be available *via* phone 24/7 to address any concerns that arise.

Safety will be assessed at weekly intervals in pre-organized Zoom meetings and at any other time as required. All suspected adverse events (SAE) will be followed up with the Committee coordinating chief investigator and all relevant medical history documented and maintained in a file of “identified” subjects.

All device issues will be directed to the device support officer. In the case of helmet failure, the faulty device will be repaired by a qualified medical devices electrical engineer, or a new device will be provided.

### Patient timeline

The schedule of clinical research activities is illustrated in Table 2. Participant investigator interaction will consist of four Zoom meetings for screening and assessment and Zoom meetings weekly for initial and ongoing device management. As well, ongoing email, telephone calls, and Zoom meetings will be provided as necessary during the period of device usage.

**TABLE 2:** Patient timeline.

Week 1: Email received at Trial office
Week 1: Zoom meeting organized for initial screening and consent
Week 2: Zoom meeting to obtain baseline assessments
Week 3: Zoom meeting for device management and treatment with PDNeuro Helmet
Week 3: Treatment commences
Weekly: Email, telephone, and Zoom meetings for ongoing device concerns as required
Week 12: Zoom meeting re-assessments
Week 12: Email and telephone call to organize return of PDNeuro Helmet
Week 12: Patients from sham group provided with active PDNeuro Helmet
Weekly: Email, telephone, and Zoom meetings for ongoing device concerns as required to second treatment group
Week 24: Zoom 6-month follow-up of all baseline assessments

### Data collection

Once accepted on the trial all data will be stored in a secure location both in paper and electronic form. Data collected will include patient’s sociodemographic data and clinical history. Next, the primary outcomes and the secondary outcomes of the study will be collected. At the end of 12 weeks, the primary and secondary outcomes of the study participants will be reassessed by the same evaluator who performed the baseline assessment. Further data will be collected following 24 weeks when Group 2 (the sham group) have also had the chance to experience the treatment and Group 1 (the initial treatment group) have had a 12-week washout. The investigators responsible for collecting all outcome measurements will be blinded to the treatment being administered to the patients.

### Statistical analysis

This is a feasibility study trialing the efficacy of the “PDNeuro” Helmet transcranial device. The sample size of 40 participants falls within the recommendation of between 12 and 50 for feasibility clinical studies (Julious, 2005; Sim and Lewis, 2012). As a series of *N* = 1 studies, it is anticipated that only basic statistical analysis will be performed. For each outcome measure, descriptive data (mean, standard deviation) will be calculated. From this data, “minimally important difference” (MID) scores will be computed based on 1/2 SD of each measure. This is a common MID measure (Norman et al., 2003) based on the distribution of the participant scores at baseline and provides a sensitive indicator of significant change over time for *N* = 1 case studies. This has also been used with good effect in previous PBMt PD trials (Liebert et al., 2021). As such, it does not suffer from a lack of statistical power that would be evident with more traditional ANOVA approaches with small sample sizes. The number of participants showing improvement (i.e., difference between two time points > MID) can be compared between time points with chi-square analyses.

In total, 162 participants (81 + 81) were proposed for recruitment over a 2-year period. However, due to limitations involving in no small part to the substantial COVID-19 restrictions during the conception of this trial, the decision was made to reduce the total number of participants to 40, with the intention of increasing this by 122 at a later date.

## Discussion

### Dissemination of results

The results addressing study objectives will be disseminated to relevant research, clinical, health services, and patient communities through requisite publications in peer-reviewed journals and presentations at scientific and clinical conferences, as well as media channels. These results will build upon safety and efficacy data to aid in the management of PD patients and in the development of a sufficiently powered larger randomized RCT.

### Advantages and limitations

This is a triple blinded RCT designed to minimize bias and maximize the validity of any differences observed between the treatment and sham groups. While the triple blinding of this RCT is an advantage, restrictions resulting from SARS-CoV-2 affected the initial protocol, creating several limitations. Due to restrictions in Australia during the conception of this trial, it was not feasible to invite participants into a clinic setting for in-person intervention and assessments due to government regulations at the time. Furthermore, the increasing rate of infections and hospitalizations during this period meant that movement of research staff and participants to and from a clinic/hospital location would introduce an increased risk of potential COVID-19 infection. Therefore, the decision was made to pivot the study to a home-based trial to minimize as much person-to-person contact as possible, and to mitigate any risk of infection to our vulnerable population.

The scientific/clinical validity and feasibility of conducting our RCT remotely had been assessed prior to commencement of the study. For example, the UPDRS assessment used for the RCT has been shown to be clinically valid when conducted remotely (Schneider et al., 2020). Indeed, the feasibility, reliability, and value of implementing remote telemedicine/video-based assessments for Parkinson’s disease patients for both research (Tarolli et al., 2020) and ongoing healthcare (Cubo et al., 2020) have been previously evaluated and deemed valid. Furthermore, all outcome measures will be assessed by medical professionals (physicians, nurses, physiotherapists) who have been trained to conduct clinical assessments in Parkinson’s disease patients, with supervision of leading neurologist to ensure assessment accuracy and reliability.

The resulting limitation is that treatment is delivered unsupervised with only a carer present. This means that there is a reliance of self-reporting and participant honesty that the “PDNeuro” Helmet is being applied correctly for the duration of the treatment period. Assessments are also being recorded *via* video communication and although cameras are effective in providing visual feedback it is possible that fine tremors may be missed and reliance of carers to manipulate the camera and give good visual observation may present a challenge.

Both motor and non-motor symptoms will be assessed using validated assessment tools for PD patients. Assessors are all trained in the delivery of each assessment tool. Furthermore, both initial and follow-up assessment are to be performed by the same assessor to increase Intrarater reliability.

Another advantage is that this study will use both quantitative and qualitative data to explore the effect of multiple signs and symptoms to evaluate efficacy of PBMt.

### Summary

This manuscript details the protocol for a prospective, single-centre study of a novel, portable LED neuro-helmet to evaluate its safety, efficacy, and tolerability. This study is important for the field of neurodegenerative disease, in particular PD, for several reasons.

The overall aging of the general population, together with the increasing prevalence of PD, means that diverse treatment options need to be explored. If the “PDNeuro” LED Helmet device is demonstrated to be safe and effective, then it potentially offers a portable, non-invasive, and inexpensive non-pharmaceutical treatment modality with minimal side effects that can be conveniently administrated at home or in an office/clinic.

## Data availability statement

The original contributions presented in this study are included in the article/supplementary material, further inquiries can be directed to the corresponding author.

## Ethics statement

The studies involving human participants were reviewed and approved by Sydney Adventist Health (SAH) Human Ethics Research Committee (HREC), approval number (2019-032). The patients/participants will provide their written informed consent to participate in this study. Written informed consent will be obtained from the individual(s) for the publication of any potentially identifiable images or data included in this article.

## Author contributions

AL and HK: conceptualization. CM: writing – original draft preparation. All authors contributed to the article and approved the submitted version.
